# Molecular prevalence and associated risk factors of *Cryptosporidium* spp. infection in dairy cattle in Khon Kaen, Thailand

**DOI:** 10.14202/vetworld.2024.371-378

**Published:** 2024-02-15

**Authors:** Bamphen Keomoungkhoun, I Putu Gede Yudhi Arjentinia, Somboon Sangmaneedet, Weerapol Taweenan

**Affiliations:** Division of Pathobiology, Faculty of Veterinary Medicine, Khon Kaen University, Khon Kaen 40002, Thailand

**Keywords:** bovine cryptosporidiosis, *Cryptosporidium*, dairy cattle, molecular prevalence

## Abstract

**Background and Aim:**

*Cryptosporidium* spp. are important parasites in the small intestines of humans and animals, particularly cattle. The aim of this study was to estimate the molecular prevalence and associated risk factors of *Cryptosporidium* infection in dairy cattle in five districts of Khon Kaen province, Thailand, and to identify *Cryptosporidium* spp.

**Materials and Methods:**

From July 2020 to October 2021, 296 fecal samples were collected from three groups of dairy cattle: Calves aged <3 months, calves aged 3 months–1 year, and calves aged >1 year. *Cryptosporidium* spp. were detected by polymerase chain reaction (PCR) amplifying the 18s RNA gene. Both genus-specific and species-specific primers were used to identify *Cryptosporidium* confirmed by DNA sequencing. Age, house floor type, and water trough type were evaluated as risk factors. We analyzed all associated risk factor information using the logistic regression test in the Statistical Package for the Social Sciences.

**Results:**

PCR results showed that 40 (13.51%) out of 296 samples were positive for *Cryptosporidium* spp., including *Cryptosporidium bovis* (57.50%) and *Cryptosporidium ryanae* (2.50%). There was a significant association between *Cryptosporidium* incidence, cattle age, and house floor type (p < 0.05). National Center for Biotechnology Information Basic Local Alignment Search Tool displayed 99.48%–100% nucleotide similarity of each *Cryptosporidium* spp. isolate with references recorded on GenBank.

**Conclusion:**

*C. bovis* and *C. ryanae* are commonly found in dairy cattle, especially calves, in Khon Kaen, Thailand, and the incidence was associated with age and house floor type. A molecular technique may be influential for species identification. The results of the present study would provide useful information for veterinarians and animal owners to understand better *Cryptosporidium* spp. and how to manage farms properly.

## Introduction

Cryptosporidiosis, caused by the protozoan *Cryptosporidium* spp., is accepted as zoonosis and causes economic loss and health problems in both humans and animals [1]. Hosts become infected by ingestion of sporulated oocyst-contaminated food or water [2]. *Cryptosporidium* spp. infection has been reported in cattle, sheep, goats, horses, and deer [3]. Diarrhea is a significant symptom, particularly in young animals. The prevalence of *Cryptosporidium* infection in neonatal calves was high at 93%, reaching up to 38% of infected calves with watery diarrhea [4]. The main symptoms of infected calves include severe diarrhea, dehydration, growth retardation, and sometimes death [5].

More than 20 species of *Cryptosporidium* have been reported in cattle. However, four are seriously pathogenic; *Cryptosporidium parvum*, *Cryptosporidium bovis*, *Cryptosporidium ryanae*, and *Cryptosporidium andersoni* are accepted [5–9]. The morbidity rate associated with *Cryptosporidium* spp. can become a serious problem depending on the risk factors. For example, the prevalence of *Cryptosporidium* spp. infection is reportedly related to age. There is a significantly high prevalence in calves below 1 month of age [10]. The rainy season has also been associated with the prevalence of *Cryptosporidium* spp. infection [11]. The polymerase chain reaction (PCR) technique has been developed for the identification of parasitic species, together with the microscopic examination for diagnosis. PCR has high sensitivity and specificity for disease diagnosis, especially in the case of protozoa oocysts that are very similar and difficult to observe under the microscope [12].

There are only a few reports on the molecular prevalence of *Cryptosporidium* spp. infection in cattle in Thailand. For example, a study in Ratchaburi and Kanchanaburi province demonstrated the prevalence of *Cryptosporidium* infection in cattle aged 2 months to >4 years by PCR-restriction fragment length polymorphism (PCR-RFLP) [13]. In a district of Khon Kaen province, the prevalence of *Cryptosporidium* infection in neonatal calves aged 1–28 days was 21% by nested PCR [6].

However, the prevalence of *Cryptosporidium* infection in other age groups of cattle, including calves, weaners, and adults, especially in other districts of Khon Kaen province, has not been estimated before. Moreover, information on the molecular prevalence of cryptosporidiosis is more necessary and useful for future treatment and control. Therefore, the aim of this study was to estimate the molecular prevalence and associated risk factors of *Cryptosporidium* infection in dairy cattle in five districts of Khon Kaen province, Thailand, and to identify *Cryptosporidium* spp.

## Materials and Methods

### Ethical approval

The Institutional Animal Care and Use Committee of Khon Kaen University approved this study (reference no. 660201.2.11/646 (121).

### Study period and location

**Figure-1:**
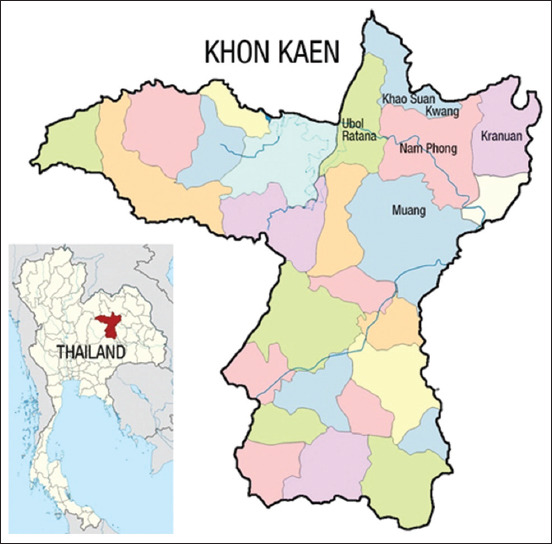
Location of research area (source: https://en.wikipedia.org/wiki/Khon_Kaen_province).

The present study was carried out from July 2020 to October 2021 in smallholder dairy cattle farms in five districts of Khon Kaen province: Muang, Namphong, Ubolratana, Kranaun, and Kaosaunkwang (Figure-1). Khon Kaen is located in the northeastern part of Thailand at a latitude of 16°26′N and longitude of 102°50′E. The main occupation of some people in these areas is to raise dairy cattle, while rice farming is the main occupation of most. These farms are approximately 1–5 km from each other within each district studied.

### Sample size

The sample size was based on 51% *Cryptosporidium* spp. infection among dairy calves under 28 days of age in Khon Kaen province [6] and a 6% error allowable. Thus, the minimum sample size was 267 samples.

### Sample collection

Fecal samples were collected from 43 dairy cattle farms in five districts of Khon Kaen province.

Regardless of health status, 296 fecal samples were collected directly from the rectum of three groups of dairy cattle: Calves aged <3 months, calves aged 3 months–1 year, and cattle aged >1 year. For further molecular investigation, samples were placed in plastic bags, kept in an icebox, and taken to the Laboratory at the Faculty of Veterinary Medicine, Khon Kaen University.

Individual data, including age, water trough type, and house floor type of dairy cattle, were collected for risk factors analysis. Because all cattle in the current study were females, sex was excluded as a risk factor.

### DNA extraction

DNA was extracted from all fecal samples using a commercial kit (GF-1 soil sample DNA extraction kit, Vivantis, Shah Alam, Malaysia) according to the manufacturer’s instructions. Briefly, 0.5 g of glass beads, 250 mg of fecal sample, and 1 mL of SL1 buffer (Vivantis) were added into a 2-mL microcentrifuge tube. The mixtures were vortexed for 5 min, incubated at 72°C for 10 min, and then vortexed twice. DNA was eluted with 100 μL elution buffer, left for 2 min, centrifuged at 10,000× *g* for 1 min, transferred to a new 1.5 mL microcentrifuge tube, and stored at 20°C until PCR analysis.

### PCR amplification

*Cryptosporidium* was identified by nested PCR in a T100 PCR thermocycler (Bio-Rad, UK). First, primary PCR was performed for amplifying a fragment of 1325 bp of the *18S rRNA* gene using two primers AL 1687 (5′-TTC TAG AGC TAA TAC ATG CG-3′) and AL 1691 (5′-CCC ATT TCC TTC GAA ACA GGA-3′). The first 25 μL PCR reaction consisted of 12.5 μL Taq master mix (Vivantis), 0.5 μL of each primer, 0.1975 μL of Taq DNA polymerase, 10.8025 μL of PCR water, and 0.5 μL of DNA template. The samples were subjected to an initial denaturation step at 94°C for 3 min, followed by 35 cycles of 94°C for 45 s, 55°C for 45 s, 72°C for 1 min, and a final extension step at 72°C for 5 min.

A fragment of 830 bp of the primary PCR product was amplified using two primers AL 1598 (5’-GGA AGG GTT GTA TTT ATT AGA TAA AG-3’) and AL 3032 (5’-AAG GAG TAA GGA ACA ACC TCC A-3’). The PCR conditions in this step were identical to those in the first reaction, except that the annealing temperature was 59°C for 45 s. The 25 μL of PCR mixture consisted of 12.5 μL master mix, 0.5 μL of each primer, 0.1975 μL of Taq DNA polymerase, 10.8025 μL of PCR water, and 0.5 μL of amplicon from the first procedure as DNA template [14, 15].

### Identification of *Cryptosporidium* spp.

PCR was performed to identify *Cryptosporidium* spp. using specific forward and reverse primers that amplified the *18s rRNA* gene (Table-1) [14]. The 25 μL reaction mixture consisted of 12.5 μL master mix, 0.5 μL of each forward and reverse primers, 0.1975 μL of Taq DNA polymerase, 10.8025 μL of PCR water, and 0.5 μL of the second nested PCR product. The PCR conditions were as follows: Denaturation at 94°C for 3 min; 35 cycles of denaturation at 94°C for 45 s, annealing at 59°C for 45 s and extension at 72°C for 1 min; and a final extension at 72°C for 7 min. 5 μL of each PCR product was loaded in 1% agarose gel electrophoresis with 100 bp of the ladder for 35 min. Finally, the gel was visualized by Visafe Red Gel Satin (Vivantis) staining under ultraviolet illumination (Gel Doc XR+ Gel Documentation System, Bio-Rad, USA).

**Table-1:** Specific primers used for *Cryptosporidium* species.

Species	Primer	Direction	Primer sequence (5’–3’)	Polymerase chain reaction product (bp)
*Cryptosporidium bovis*	CbF	Forward	CTTCTTATTGGTTCTAGAATAAAAATG	241
	AL3032	Reverse	AAGGAGTAAGGAACAACCTCCA	
*Cryptosporidium parvum*	CphF	Forward	AGAGTGCTTAAAGCAGGCATA	305
	AL3032	Reverse	AAGGAGTAAGGAACAACCTCCA	
*Cryptosporidium ryanae*	CrF	Forward	TGTTAATTTTTATATACAATRaCTACGG	415
	AL3032	Reverse	AAGGAGTAAGGAACAACCTCCA	
*Cryptosporidium andersoni*	CaF	Forward	GCAAATTACCCAATCCTGAC	625
	AL3032	Reverse	AAGGAGTAAGGAACAACCTCCA	

### Sequencing and phylogenetic analysis

PCR products were sequenced from nested PCR samples using BTSeqTM (Barcode-Tagged Sequencing; CELEMICS, Seoul, Korea). The obtained sequences were compared to *Cryptosporidium* spp. references on the GenBank using Basic Local Alignment Search Tool provided by the National Institutes of Health (http://www.ncbi.nlm.nih.gov). We constructed a phylogenetic tree based on the maximum likelihood method with 1000 replicates for bootstrap analysis using the MEGA (version 11.0) software (https://www.megasoftware.net).

### Statistical analysis

The prevalence of *Cryptosporidium* infection in each age group and the relationship between associated risk factors and the prevalence were evaluated using univariate and multivariate logistic regression analysis in the Statistical Package for the Social Sciences version 19 (IBM Corp., NY, USA). p < 0.05 was considered for all statistical significance.

## Results

### Sample analysis

**Figure-2:**
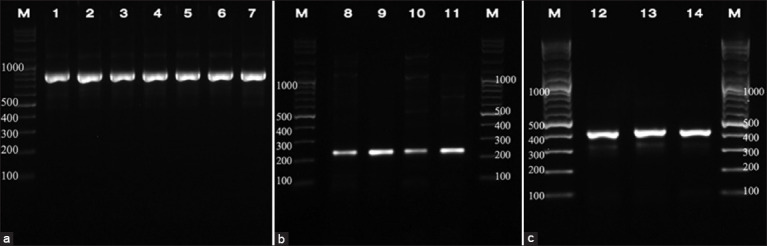
Gel electrophoresis of PCR products. (a) Lane 1–7=*Cryptosporidium* spp. (840 bp) (b) Lane 8–11=*Cryptosporidium bovis* (241 bp) (c) Lane 12–14=*Cryptosporidium ryanae* (415 bp) M=Marker (2000–100 bp). PCR=Polymerase chain reaction.

Using genus-specific primers, 13 (30.2%) out of 43 farms and 40 (13.51%) out of 296 samples from five districts were positive for *Cryptosporidium* (Table-2). Specific primers for each species were used for species identification by single PCR. Two species, *C. bovis* and *C. ryanae*, were identified: *C. bovis* (57.5%) was detected in 23 samples, but *C. ryanae* (2.5%) was detected in only one sample. In addition, 16 samples contained mixed infections of both *C. bovis* and *C. ryanae* (40%) (Figure-2).

**Table-2:** Prevalence of *Cryptosporidium* spp. in dairy cattle from five districts of Khon Kaen.

No.	Area	% Prevalence (no. of positive/no. of samples)	95% confidence interval
1	Muang	10.29 (7/68)	3.08–17.50
2	Namphong	85.00 (17/20)	69.36–100
3	Ubolratana	10.25 (12/117)	4.76–15.74
4	Kranuan	2.22 (1/45)	0.00–6.48
5	Khaosuankwang	6.52 (3/46)	0.00–13.65
Total		13.51 (40/296)	9.22–17.80

### Prevalence and associated risk factors of *Cryptosporidium* spp.

In the present study, *Cryptosporidium* spp. prevalence was significantly associated with cattle age (p < 0.05). The risk of *Cryptosporidium* infection was higher in <3-month-old calves (odds ratio [OR] = 12.13, 95% confidence interval [CI]: 4.58–32.14) than in adults (>1 year old) (Table-3).

**Table-3:** *Cryptosporidium* spp. prevalence and associated risk factors.

Factor	% Prevalence (no. of positive/no. of samples)	Univariable analysis		Multivariable analysis	
	
		Odds ratio (95% CI)	p-value	Adjusted odds ratio (95% CI)	p-value
Age					
>1-year-old	5.60 (7/125)	1		1	
3-month–1-year-old	11.71 (15/128)	2.23 (0.87–5.59)	0.11	2.24 (0.87–5.77)	0.09
<3-month-old	41.86 (18/43)	12.13 (4.58–32.14)	0.00001**	10.41 (3.85–28.11)	0.00001**
House floor type					
Concrete	7.52 (14/186)	1		1	
Ground	23.63 (26/110)	3.73 (1.85–7.52)	0.0002**	3.12 (0.98–9.94)	0.054
Water trough type					
Moveable container	9.85 (14/142)	1		1	
Concrete tank	16.88 (26/154)	1.85 (0.92–3.71)	0.08	1.16 (0.36–3.76)	0.79

CI=Confidence interval;

** Statistically significant difference (p≤0.01)

House floor type was significantly associated with *Cryptosporidium* prevalence (p < 0.05). Cattle housed on the ground floor had a higher risk (OR, 3.73; 95% CI, 1.85–7.52) compared with cattle housed on the concrete floor. Concurrently, there was no association between *Cryptosporidium* infection and water trough type (p > 0.05), although the prevalence of *Cryptosporidium* spp. tended to be higher in cattle raised on farms using concrete tanks (OR = 1.85, 95% CI: 0.92–3.71) than in cattle raised on farms using moveable containers (Table-3). According to three factors, age was significantly associated with a higher risk of infection (p < 0.05) compared with house floor type and water trough type using multivariable analysis (OR = 10.41, 95% CI: 3.85–28.11).

### Sequencing and phylogenetic tree analysis

**Figure-3:**
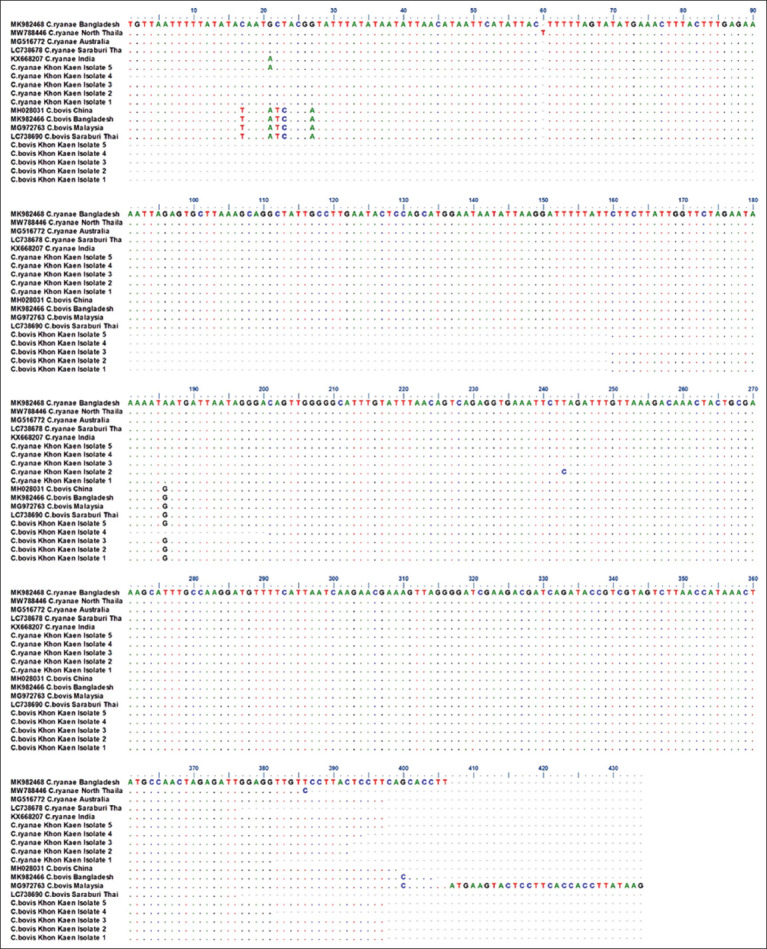
Alignment of the local sequences obtained from 18S rRNA gene of each *Cryptosporidium bovi*s and *Cryptosporidium ryanae* from this study and some reference sequences from GenBank. Dots (.) denote the identical nucleotides.

Based on clear bands on agarose electrophoresis gels, five of 39 positive *C. bovis* samples and five of 17 positive *C. ryanae* samples were randomly selected to confirm the species identification. We aligned the sequences of the present study with some references from GenBank. Local *Cryptosporidium* spp. isolates from Bangladesh (MK982466 and MK982468), Australia (MG516772), India (KX668207), Malaysia (MG972763), China (MH028031), and Saraburi province and northern part of Thailand (LC738690, LC738678, and MW788446) were found to be closely related to National Center for Biotechnology Information *Cryptosporidium* spp. with 99.48%–100% nucleotide similarity (Figure-3).

**Figure-4:**
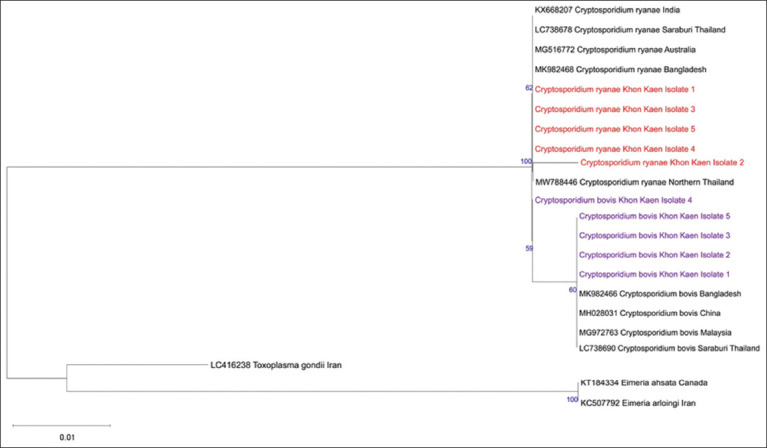
Phylogenetic tree of local *Cryptosporidium* *ryanae* (red) and *Cryptosporidium bovis* (purple) Khon Kaen isolates, and the references recorded on GenBank. The phylogenetic tree was constructed using the maximum likelihood method in MEGA 11 software. Numbers indicate the bootstrap percentages from 1000 replicates.

According to the phylogenetic tree analysis results, the local *C. ryanae* Khon Kaen isolates 1, 3, 4, and 5 were closely related to each other and matched with some references recorded in GenBank, including *C. ryanae* KX668207 from India, MG516772 from Australia, MK982468 from Bangladesh, and LC738678 from Saraburi, central part of Thailand, whereas *C. ryanae* Khon Kaen isolate 2 was close to MW788446 from northern part of Thailand. *C. bovis* Khon Kaen isolates 1, 2, 3, and 5 from the present study were grouped with *C. bovis* MK982466 from Bangladesh, MH028031 from China, MG972763 from Malaysia, and LC738690 from Saraburi province, Thailand, whereas *C. bovis* Khon Kaen isolate 4 was separately isolated. Sequences of *Toxoplasma gondii* (LC416238), *Eimeria arloingi* (KC507792), and *Eimeria ahsata* (KT184334) were added as outgroups (Figure-4).

## Discussion

In the present study, we demonstrated the molecular prevalence of *Cryptosporidium* infection in cattle of different age groups in five districts of Khon Kaen province and analyzed the associated risk factors of *Cryptosporidium* infection. The overall molecular prevalence of *Cryptosporidium* spp. was 13.51% in cattle aged 1 week–7 years. The prevalence in the present study was lower than that in a previous report (21%) that focused on calves aged only 1–28 days in one district of Khon Kaen province [6]. However, the prevalence in the present study was close to that of another previous study (15.50%) in Ratchaburi and Kanchanaburi province, Thailand, which studied cattle aged <2 months to more than 4 years [13]. Moreover, the prevalence in this study was close to 13.8% (53/384) in calves in Ethiopia [16]. The prevalence of *Cryptosporidium* spp. in cattle varies across countries worldwide. For example, *Cryptosporidium* spp. were found in 46.8% in Ningxia, China [5], 16.20% in India [11], 35.70% in Vietnam [17], 33.1% in Kuwait [18], 24.4% in Taiwan [19], 5% in Iran [20], and 4.38% in Guangdong, China [21]. These data indicate the prevalence of *Cryptosporidium* in different geographical zones.

In the present study, four common species in cattle were investigated using specific primers. Only *C. bovis* and *C. ryanae* were detected in this study. This finding is consistent with a previous report showing that *C. bovis* and *C. ryanae* were mainly found in dairy calves in Khon Kaen, whereas *C. andersoni* and *C. parvum* were not detected by nested PCR [6]. However, there is no agreement with another previous study which revealed that *C. parvum* was reported in dairy cattle in western Thailand by PCR-RFLP [13]. Geographical distribution may be a factor that affects the different species found [22].

The prevalence of *Cryptosporidium* spp. in this study was significantly associated with the age of cattle according to risk factor analysis. The prevalence of *Cryptosporidium* was significantly higher in calves <3 months old (41.86%) than in cattle more than 1 year old (5.60%) (OR = 12.13, 95% CI: 4.58–32.14, p = 0.00001). Our results are consistent with those of a previous study, which showed that calves younger than 1 month (19.30%) were more likely to become infected with *Cryptosporidium* spp. than those between 1 and 3 months (10.90%) [11]. Another study demonstrated that *Cryptosporidium* spp. (16.30%) was more prevalent in calves <3 months than in weaned calves (3.30%) and adults (1.40%) [23]. This is due to the poor immunity of calves in the first infection compared to older ones in the reinfection [24]. To prevent infection, calves should be raised in a hygienic pen and kept separately from adult cattle.

The present study demonstrated an association between *Cryptosporidium* spp. infection and house floor type. Cattle housed on the ground had a significantly higher risk of *Cryptosporidium* spp. infection compared with cattle housed on the concrete floor (OR = 3.73, 95% CI: 1.85–7.52, p = 0.0002). This finding is similar to a previous report showing that the risk of infection was higher in calves housed with straw or earth floors (OR = 1.6) than in calves housed in pens with concrete floors [23]. This finding is in agreement with another previous study that demonstrated a significant difference in infection between calves housed on a dirt floor (OR = 18.4) compared to calves housed on a cement floor [24]. This is because the ground is more difficult to clean than the concrete floor; thus, oocysts will not be eliminated, and animals still have the opportunity to contact them [25]. As a result, owners should be advised to keep animals, particularly calves, on a concrete floor to reduce infection.

Although water trough type was not significantly associated with *Cryptosporidium* spp., infection in cattle raised on farms using concrete tanks as drinking water containers tends to increase the risk of infection. Due to the difficulty of removing the stagnant water and cleaning the concrete tank, there is an increased risk of contaminated *Cryptosporidium* spp. oocysts in drinking water, thereby increasing *Cryptosporidium* spp. infection. The prevalence of *Cryptosporidium* infection was 11.78 (95% CI: 66–61.5) times higher in farms with stagnant water than in farms without stagnant water (p = 0.05) [26]. Limited access to drinking water and river/stream drinking water sources was also associated with *Cryptosporidium* infection [27].

The present results of phylogenetic tree analysis indicated that *C. bovis* and *C. ryanae* Khon Kaen isolates had 99.48%–100% homology to the reference sequences recorded on GenBank. The current findings are consistent with previous reports showing that *C. bovis* and *C. ryanae* are closely related and not distant from each other [22, 28–31]. In addition, the local Khon Kaen isolates were homologous to the reference isolates from other parts of Thailand.

However, this study had limitations. For example, more cattle and farms could be collected with a similar number of samples in each age group in each area. Further, investigation could be performed to increase the accuracy of the incidence of *Cryptosporidium* infection. Other risk factors, such as the number of animals per pen, humidity conditions of the pen, hygiene management system, or seasonal effects, could also be considered in this study.

## Conclusion

The molecular prevalence of *Cryptosporidium* spp. in dairy cattle in Khon Kaen was 13.51%. Interestingly, *C. parvum*, considered an important zoonotic pathogen, and *C. andersoni* were not detected in the studied areas, whereas two species, *C. bovis* (57.50%) and *C. ryanae* (2.50%), were identified. Associations were observed between *Cryptosporidium* infection and age as well as house floor type (p < 0.05). This survey provided information on the molecular prevalence, genetic identification, and associated risk factors of *Cryptosporidium* spp. that could enhance the best control and prevention of bovine cryptosporidiosis in this region.

## Authors’ Contributions

BK: Data curation, investigation, formal analysis, and writing original manuscript. BK and IPGYA: Sample collection, methodology, and resources. WT and SS: Resources, supervision, and validation. WT: Conceptualization, project administration, review, and editing manuscript. All authors have read, reviewed, and approved the final manuscript.
